# Metal–Metal Bonding Process Research Based on Xgboost Machine Learning Algorithm

**DOI:** 10.3390/polym15204085

**Published:** 2023-10-14

**Authors:** Jingpeng Feng, Lihua Zhan, Bolin Ma, Hao Zhou, Bang Xiong, Jinzhan Guo, Yunni Xia, Shengmeng Hui

**Affiliations:** 1 State Key Laboratory of Precision Manufacturing for Extreme Service Performance, Central South University, Changsha 410083, China; fengjingpeng4@gmail.com (J.F.); yjs-cast@csu.edu.cn (L.Z.); 233812049@csu.edu.cn (H.Z.); xiongbang8638@163.com (B.X.); guruci@163.com (J.G.); xyn13467535590@163.com (Y.X.); 223801010@csu.edu.cn (S.H.); 2 Light Alloys Research Institute, Central South University, Changsha 410083, China

**Keywords:** single-lap joints, finite element models, Xgboost machine learning algorithm, interpretation toolkit SHAP, process parameter optimization

## Abstract

Conventionally, the optimization of bonding process parameters requires multi-parameter repetitive experiments, the processing of data, and the characterization of complex relationships between process parameters, and performance must be achieved with the help of new technologies. This work focused on improving metal–metal bonding performance by applying SLJ experiments, finite element models (FEMs), and the Xgboost machine learning (ML) algorithm. The importance ranking of process parameters on tensile–shear strength (TSS) was evaluated with the interpretation toolkit SHAP (Shapley additive explanations) and it optimized reasonable bonding process parameters. The validity of the FEM was verified using SLJ experiments. The Xgboost models with 70 runs can achieve better prediction results. According to the degree of influence, the process parameters affecting the TSS ranked from high to low are roughness, adhesive layer thickness, and lap length, and the corresponding optimized values were 0.89 μm, 0.1 mm, and 27 mm, respectively. The experimentally measured TSS values increased by 14% from the optimized process parameters via the Xgboost model. ML methods provide a more accurate and intuitive understanding of process parameters on TSS.

## 1. Introduction

Due to the benefits of obtaining lighter and stronger structures with better weight reduction, smaller stress concentrations, more uniform stress distribution, and lower manufacturing costs compared to traditional metal connections (such as welding, bolting, and riveting) [1,2,3], adhesive bonding technology is widely used in the aerospace, automotive, marine, wind energy, construction, and furniture fields [4]. Besides the above advantages, adhesive bonding technology for metal–metal structures also has excellent fatigue resistance and damage tolerance properties, making it beneficial for aerospace application [5].

Single-lap joints (SLJs), stepped-lap joints, double-lap joints, scarf joints, tube joints, and butt strap lap joints are common examples [6]. Among them, SLJs are representative and have attracted wide attention from scholars. For instance, Pinto et al. [7] evaluated the effect of different adhesive thicknesses on the tensile strength of metal–metal SLJs bonded with ductile and brittle adhesives by numerical and experimental methods. Rodríguez et al. [8] modified several SLJ stress models for Volkersen, Goland and Reissner, Hart-Smith, and Ojalvo and Eidinoff. Zimmermann et al. [9] developed an analytical model to describe the out-of-plane deformation at the end of the lap joint.

Furthermore, researchers are also concentrating on understanding the damage behavior of bonded joints and adhesive failure modes. Grant et al. [10] investigated the effect of adhesive thickness change on the bending moment at the edge of metal–metal joints and proposed a joint failure criterion. Natu et al. [11] stated that adhesives exist in adhesive failure, adhesive/cohesive failure, cohesive failure, and matrix failure modes with metal–metal joints. Cui et al. [12] investigated the effects of surface roughness, lap length, and adhesive layer thickness on the shear strength of metal–metal SLJs with experimental methods, and they further explored the damage failure modes of different bonded joints. Meanwhile, the interface between the adhesive and the adherend becomes the concern of the research, and interfacial bonding is affected by wettability, interfacial defects, and other factors [13]. Guo et al. [14] emphasized that anodization can introduce nanoscale porous structures to the metal surface, increase the wettability between the metal and adhesive, and further improve interfacial bonding between metal and adhesive. The reliability and performance of bonded joints will deteriorate due to grease on the interface [15]. These research studies are essential in promoting broader engineering applications of metal–metal bonding technology.

Moreover, the metal–metal joint connection needs to be parametrically designed for better bonding qualities; an important indicator is the shear strength of the joints [16]. The orthogonal experiment method is frequently used to optimize the process parameters. However, there is a disadvantage of fewer sample data and a large level span of experimental design, resulting in optimized process parameters that may not be optimal. More sample data can be obtained by the finite element models (FEMs), and Zhang et al. [17] utilized the FEM to generate large amounts of virtual data as a data source for machine learning (ML). In recent years, ML has been a promising technology with increasingly obvious advantages in data model fitting and data mining, through utilizing a large amount of accumulated experimental data, improving the efficiency of new material design and development, and significantly reducing the consumption of funds and time. Meanwhile, as a powerful tool to reveal and characterize the complex relationship between material properties and individual features, Jeon et al. [18] evaluated the importance ranking of tempering temperature, holding time, and various elements on Vicker’s hardness by using the ML algorithm and interpretation toolkit SHAP (Shapley additive explanations). Compared to traditional physical methods, ML can directly face the required properties by optimizing the manufacturing process of existing materials, avoiding the intertwined processes of physics and chemistry [19]. ML has been successfully applied in materials science fields [20,21].

As one of the ML boosting models, the Xgboost model improves prediction accuracy by controlling model complexity and reducing variance. Therefore, there is a lower risk of overfitting and a simpler trained model. Calculating each feature’s marginal contribution to the model’s output is the main goal of SHAP, and this method’s biggest benefit is that it makes it clear whether the input’s known conditions have a positive or negative impact on the prediction’s results, thus avoiding the “black box” problem of other ML models [22]. However, there are only a few applications of ML models for predicting the failure of bonded joints. Gu et al. [23] applied a deep neuronal network (black box) coupled with a genetically programmed (gray box) model for predicting the failure loads of metal–composite bonded joints. As far as we know, there is no published literature for predicting the mechanical performance of metal–metal single-lap specimens using the Xgboost ML model. Therefore, it provides an opportunity for this work.

In this work, the tensile–shear strength (TSS) of metal–metal SLJs was investigated. Firstly, the validity of the FEM was verified by single-lap process experiments. Secondly, considering the effects of different process parameters such as lap length (LL), the thickness of the adhesive layer (TAL), and roughness, 630 sets of single-lap FEMs were established, the maximum load of each model was extracted, and the TSS was calculated. A total of 630 sets of TSS data were obtained with different process parameters. Finally, 504 sets of training set data were used to train the Xgboost model, and 126 sets of test set data were used to predict TSS. The importance ranking of input features on model outputs was obtained with the interpretation toolkit SHAP. The reasonable bonding process parameters were optimized. ML methods provide a more accurate and intuitive understanding of process parameters on TSS.

## 2. Materials and Methods

### 2.1. Material Pretreatment

**Figure 1:**
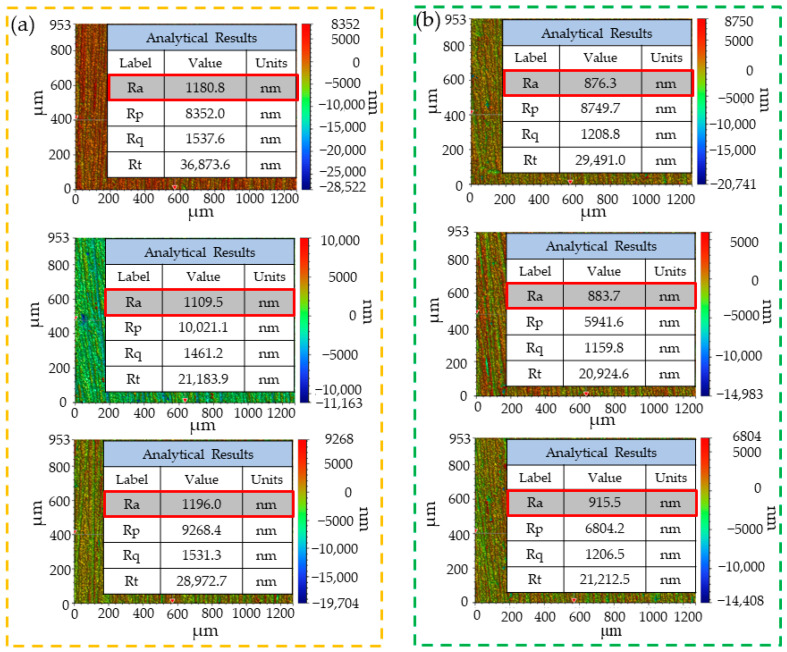

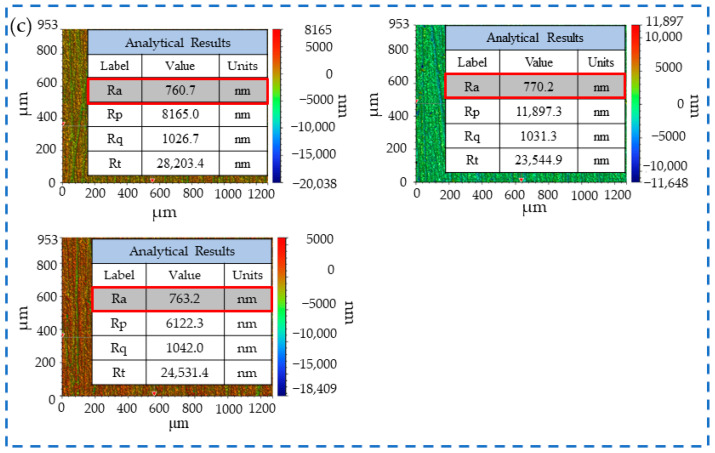
Metal surface roughness values: (**a**) Type-1; (**b**) Type-2; (**c**) Type-3.

As the literature [24,25] suggested, the metal lap areas should be polished and anodized before metal–metal lapping. In this work, the metal surface was pretreated by phosphoric acid anodizing after sanding with 400#, 800#, and 1200# sandpaper. The surface roughness was measured with a MyKo NT9100 optical surface profiler, as shown in Figure 1. The metal surface pretreatment process was shown in Table 1.

In addition, the sanding direction was perpendicular to the length of the adherend until there were visible sanding scratches. The anodizing process includes:

(1) Firstly, 25–30 g/L NaOH solution was degreased for 2 min.
(2) Secondly, 300 g/L HNO_3_ solution was pickled for 1 min.
(3) Thirdly, 150 g/L H_3_PO_4_ solution was anodized for 20 min, and the voltage value was stabilized at 15 V.

### 2.2. Curing Process

**Figure 2:**
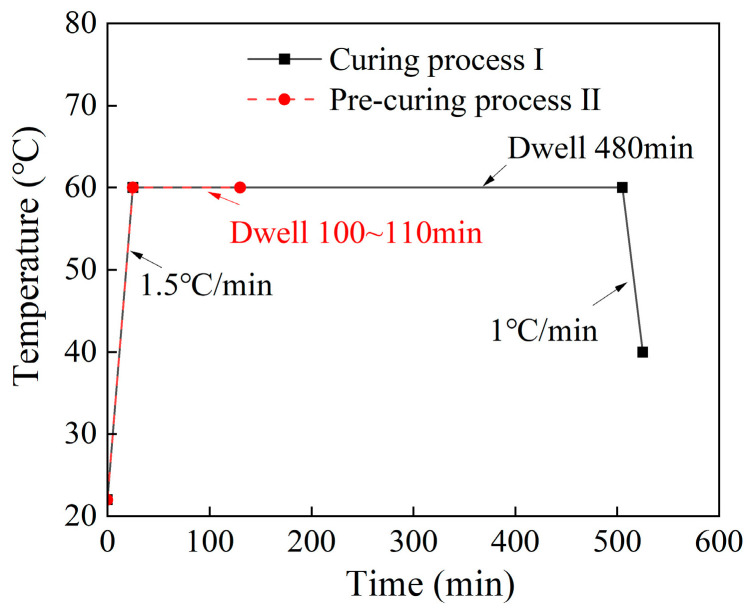
Curing process of DW-3 adhesive.

The adhesive DW-3 resin produced by Shanghai Huayi Resin Co., Ltd. (Shanghai, China) was applied in this work. It was mixed with A, B, and C components according to the weight ratio of 5:1:0.2. Among them, Component A is an epoxy polymer, Component B is an aromatic amine synthesizer, and Component C is a KH550 silane coupling agent, and its function improves the interfacial interaction between inorganic and organic substances. The vacuum mixing defoamer, typed as TMV-200T, was used to mix more uniformly and reduce bubbles. Based on the autoclave platform, the curing process I for the DW-3 adhesive involved heating to 60 °C at 1.5 °C/min and then holding for 480 min, followed by cooling down to 40 °C at a rate of 1 °C/min (shown in Figure 2). In conventional cases, the DW-3 adhesive has good flowability, and it is easy for it to seep out from the edge of the overlap to form gel tumors [26,27], which will increase the bonding area and make the TSS be calculated inaccurately through Equation (1).

### 2.3. SLJ Specimen Curing

**Figure 3:**
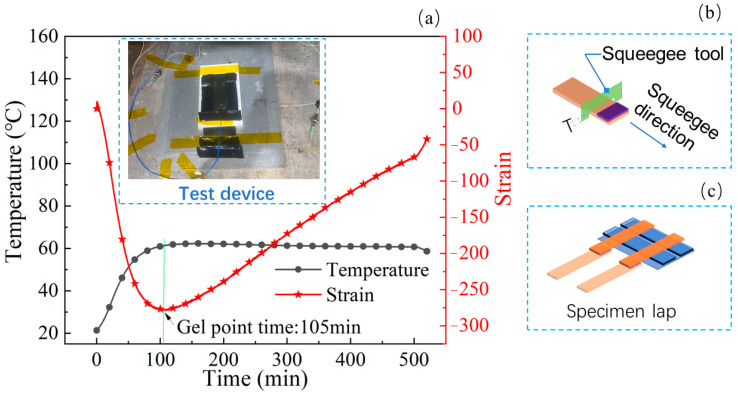
The diagram of the GP acquisition device, squeegee tool, and specimen lap. (**a**) GP acquisition device; (**b**) squeegee tool; (**c**) specimen lap.

In this work, for the purpose of improving the seepage phenomenon of the adhesive layer from the edges during the lapping process, the curing of the SLJ specimen was divided into two steps. In the first step, the pre-curing process II was determined based on the adhesive’s gel point (GP) time range. GP indicates the resin’s transition from a visco-fluid state to a highly elastic state, thus weakening its flowability. In the second step, pre-curing was conducted, followed by lapping (shown in Figure 3c) to finish the secondary complete curing (SCC). The pre-curing process (PCP) dramatically improves the seepage phenomenon of resin from the lapping edges after SCC.

#### 2.3.1. Adhesive PCP Determination

Fiber Bragg grating (FBG) and thermocouples were applied to record wavelength and temperature changes during the adhesive curing process. The principle was that the change in the temperature field during the curing process would cause a change in the grating’s effective refractive index, ultimately reflected as a shift in the central reflection wavelength. Therefore, the strain acting on the grating can be calculated through changes in temperature and central reflection wavelength, and the strain calculation equation was reported in the literature [28]. It can be found that the GP of the adhesive appeared in 105 min (shown in Figure 3a). The adhesive was heated to 60 °C for 27 min at a heating rate of 1.5 °C/min and then kept in the autoclave platform for 78 min. There was no obvious seepage phenomenon of the adhesive layer at the edge of the SLJ specimen after SCC via appropriately extending the holding time from 78 min to 100~110 min. Therefore, the pre-curing process II was determined as heating to 60 °C at a rate of 1.5 °C/min with a holding time of 100~110 min.

#### 2.3.2. Pre-Curing

The adhesive is coated in the lap area of each adherend and a squeegee tool (seen in Figure 3b) is used to control the adhesive thickness; the symbol T (2 mm thick adherend +1/2 TAL) represents the groove height of the squeegee tool. With the pre-curing of the adhesive with pre-curing process II, the adhesive can fully impregnate the adherend and keep good adhesion after PCP.

#### 2.3.3. Secondary Complete Curing

**Figure 4:**
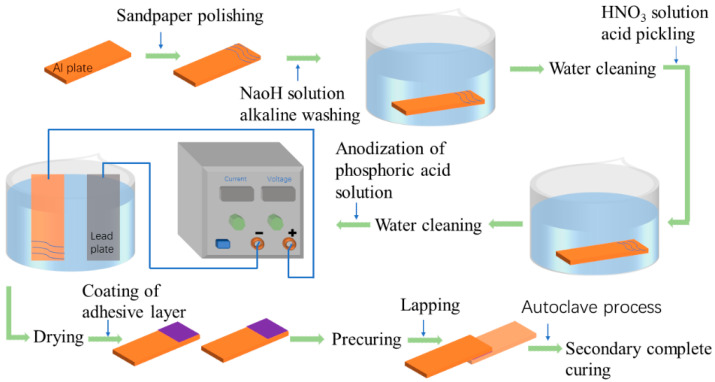
The whole process of the bonding experiment.

The lapping specimens must be vacuum-bagged, adopting curing process I to finish SCC for preparing SLJ specimens, and a 0.1 MPa vacuum is pumped based on the autoclave platform during the SCC process. The whole process of the bonding experiment is shown in Figure 4.

### 2.4. Mechanical Performance Test

**Figure 5:**
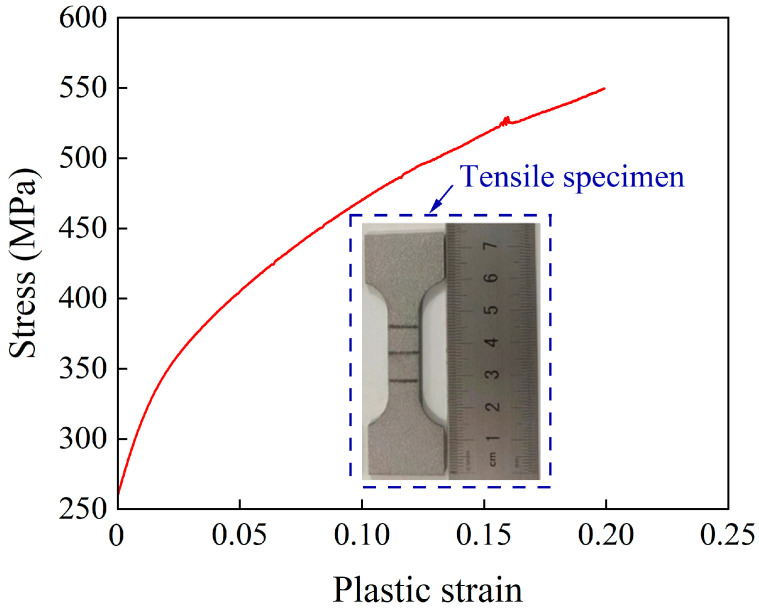
Real strain–stress curve of 2A12-T4 aluminum alloy.

A 2A12-T4 aluminum alloy produced by Shanghai Chengzhong Metal Co., Ltd. (Shanghai, China) was used in this work. The mechanical properties were measured according to the People’s Republic of China’s metal tensile test standard GB/T228.1-2010 [29] and are listed in Table 2. Nominal stress–strain data were transformed into real stress–strain data by the equation in the literature [30]. The plastic strain–stress curve of the 2A12-T4 aluminum alloy is shown in Figure 5.

In order to clarify the effect of different roughnesses on the bonding performance, the test standards ASTM-D897-08 [31] and ASTM-D1002-10 [32] were applied to measure the mechanical parameters of the adhesives, and the results are listed in Table 3.

**Figure 6:**
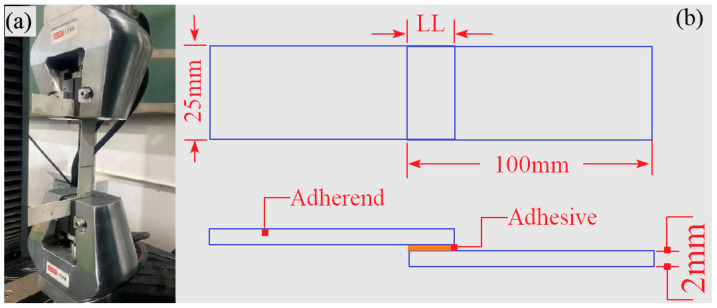
Specimen geometry size and tensile shear test device. (**a**) Tensile shear test device; (**b**) specimen geometry size.

The size of the single-lap specimen is diagramed in Figure 6b, and the TSS of the SLJs was tested according to the National Standard of the People’s Republic of China (GB7124-1986 [33]) by using the MTS universal experiment machine (shown in Figure 6a). The tensile rate was 5 mm/min, and the TSS value was calculated based on Equation (1).

*τ* = *P*/(*B* × *L*) (1)

where *τ* represents the TSS of the adhesive; symbol *P* is the maximum load for the shear failure of the specimen; and *B* and *L* indicate the width and length of the overlapping surface, respectively.

## 3. Constitutive Model

### 3.1. Elasto-Plasticity Constitutive Model [34]

The isotropic elastic constitutive equations of metals are shown in Equations (2) and (3), and the plastic constitutive equations are shown in Equations (4)–(6).

(2) $$\left[\begin{array}{c} \varepsilon_{11}\\ \varepsilon_{22}\\ \varepsilon_{33}\\ \gamma_{12}\\ \gamma_{13}\\ \gamma_{23} \end{array}\right]=\left[\begin{array}{cccccc} 1/E & -\mu /E & -\mu /E & 0 & 0 & 0\\ -\mu /E & 1/E & -\mu /E & 0 & 0 & 0\\ -\mu /E & -\mu /E & 1/E & 0 & 0 & 0\\ 0 & 0 & 0 & 1/G & 0 & 0\\ 0 & 0 & 0 & 0 & 1/G & 0\\ 0 & 0 & 0 & 0 & 0 & 1/G \end{array}\right]\left[\begin{array}{c} \sigma_{11}\\ \sigma_{22}\\ \sigma_{33}\\ \sigma_{12}\\ \sigma_{13}\\ \sigma_{23} \end{array}\right]$$

*G* = *E*/[2 × (1 + *μ*)] (3)

where *E* represents the Young’s modulus; *μ* is the Poisson’s ratio, 0.33; and *G* indicates the shear modulus.

(4) $$\sigma_{e}={\left[\frac{3}{2}\left(\sigma_{11}^{2}+\sigma_{22}^{2}+\sigma_{33}^{2}+{2\sigma }_{12}^{2}+{2\sigma }_{23}^{2}+{2\sigma }_{31}^{2}\right)\right]}^{1/2}$$

(5) $$f=\sigma_{e}-\sigma_{y}\left({d\varepsilon }_{p}\right)=0$$

(6) $$d\varepsilon_{ij}^{p}=d\lambda \sigma_{ij\;}^{\prime }$$

where $\sigma_{e}$ represents the equivalent stress; *f* expresses a function of the yield surface; $\sigma_{y}$ is the yield stress and is expressed as a function of the equivalent plastic strain; *d*$\varepsilon_{ij}^{p}$ is the equivalent plastic strain increment; and λ is the effective Lame constant.

### 3.2. The Initial Damage Model for a Cohesive Element [34]

A single-lap FEM was used to simulate the SLJ mechanical properties by applying a typical bilinear model (traction–separation law), and the characterized parameters mainly contain the modulus, critical traction force, and fracture energy (shown in Table 3). The initial damage criterion of the cohesive unit adopts the quadratic nominal stress criterion (seen in Equation (7)), and the damage begins when the sum of squared stresses of the cohesive unit in the normal, tangential 1, and tangential 2 directions reaches 1.

(7) $${\left\{\frac{{〈t}_{n}〉}{t_{n}^{0}}\right\}}^{2}+{\left\{\frac{t_{s}}{t_{s}^{0}}\right\}}^{2}+{\left\{\frac{t_{t}}{t_{t}^{0}}\right\}}^{2}=1$$

where $t_{n}$, $t_{s}$, and $t_{t}$ represent the stresses of the cohesive unit in the normal direction, tangential direction 1, and tangential direction 2, respectively. $t_{n}^{0}$, $t_{s}^{0}$, and $t_{t}^{0}$ are the maximum critical stresses of the cohesive unit in the normal direction, tangential direction 1, and tangential direction 2, respectively.

### 3.3. The Damage Evolution Model for a Cohesive Element [34]

**Figure 7:**
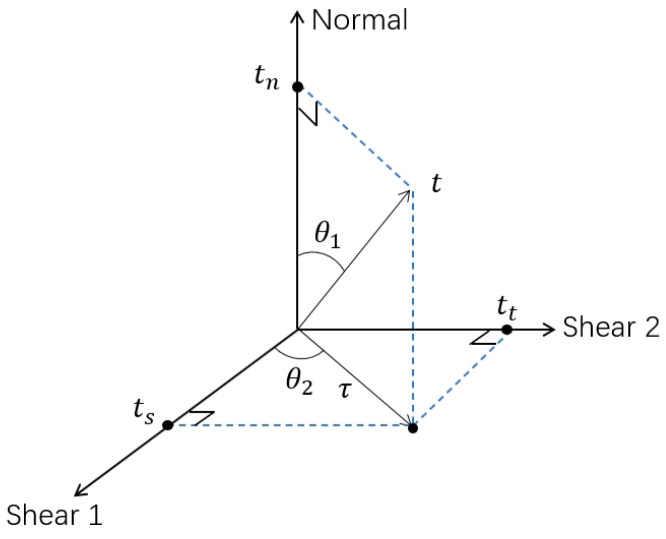
Mode mix measures based on traction.

The damage evolution stage begins after the initial damage to the adhesive occurs, and the damage evolution is based on the energy fracture criterion. The softening method is linear, and the linear softening specifies the linear softening stress–strain response of the linear elastic material or the linear evolution of the damage variables accompanying the deformation of the elastic-plastic material, and the mixing mode uses the BK energy criterion (shown in Equation (8)). Figure 7 describes a mode mix based on traction components.

(8) $$G^{C}=G_{n}^{C}+\left(G_{s}^{C}-G_{n}^{C}\right){\left\{G\frac{G_{S}}{G_{T}}\right\}}^{\eta }$$

where *Gn*, *Gs*, and *G_T_* denote the work conducted by the cohesive unit along the normal, tangential direction 1, and tangential direction 2, respectively; $G_{n}^{C}$,$G_{s}^{C}$, and $G^{C}$ denote the normal, tangential, and mixed fracture energy, respectively.

## 4. Results and Discussion

### 4.1. Numerical Model Verification

**Figure 8:**
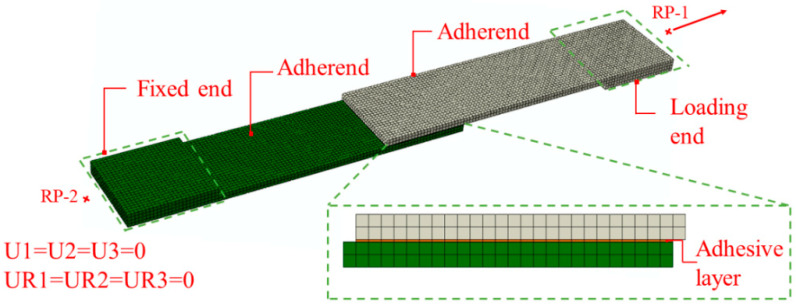
Single-lap FEM.

An LL of 25 mm, TAL of 0.2 mm, and roughness of 1.16 μm were chosen for the SLJ experiment and the establishment of the corresponding FEM. Among them, a single-lap FEM is shown in Figure 8, and a global grid size of 1 mm was applied to mesh the part. There are 6250 elements per piece of adherend, and the grid type is a C3D8R linear hexahedral element. The adhesive layer has 625 elements, and the grid type is a COH3D8 linear hexahedral element. The thickness direction is divided into grids by sweeping. The fixed end is coupled to the reference point RP-2, and the boundary conditions for RP-2 are encastre. The loading end is coupled to the reference point RP-1, and the RP-1 imposes a displacement constraint. The contact between the adhesive layer and the adherend is set as a tie.

**Figure 9:**
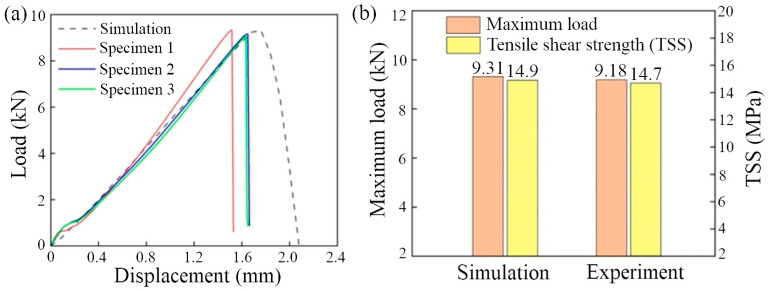
The results for the experiment and simulation of SLJs. (**a**) Load–displacement curves; (**b**) comparison of the maximum load and TSS values.

**Figure 10:**
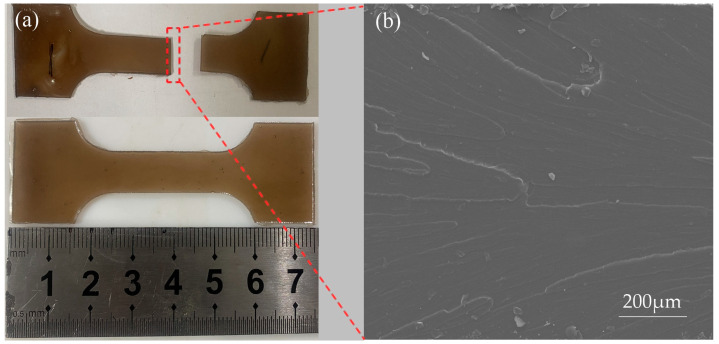
The tensile specimen fracture diagram of the adhesive: (**a**) macro; (**b**) micro.

The load–displacement curves for the experiment and simulation of SLJs during the tensile–shear process are shown in Figure 9a. The load increases with displacement, and the bonding region will rupture when the curve reaches the maximum load. That is because the cured DW-3 adhesive is a brittle material (shown in Figure 10), the tensile fracture is relatively smooth, and there is no tough nest on the fracture surface. When it reached the maximum load, the joint was peeled off, resulting in the bonding region rupture. The maximum load of the experiment and simulation curves were extracted, and the TSS values were calculated (seen in Figure 9b). The simulation results’ maximum load and TSS values are 9.31 kN and 14.9 MPa, respectively. In comparison, the experiment’s maximum load and TSS values are 9.18 kN and 14.7 MPa, respectively (the average of the three samples was taken). The relative errors of the maximum load and the TSS among the simulation and the experiment test are 1.41% and 1.36% (calculated by Equation (9)), respectively. It indicates that single-lap FEMs have a certain effectiveness.

*δ* = (*P_si_* − *P_ex_*)/*P_ex_* (9)

where parameter *δ* is the relative error, symbol *P* means the load, and the subscript index *si* and *ex* represent the simulation and experiment, respectively.

**Figure 11:**
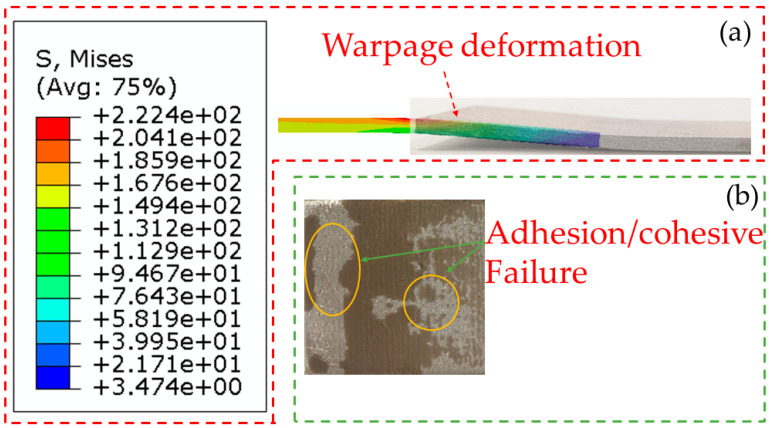
Joint failure diagram. (**a**) Simulation; (**b**) experiment.

Furthermore, the experiment and simulation demonstrate the warpage deformation in the lapping region (shown in Figure 11a). The warpage deformation can be explained by the eccentricity of the load leading to small moments at the joint ends during the tensile–shear process. When the shear strength reaches the strength of the joint, it will cause edge cracks and more significant bending moments [12,35]. Ultimately, the joint fails under the action of shear and peel stresses. The warpage degree of the two joints is almost the same, further proving the effectiveness of the single-lap FEM. Meanwhile, the adhesive is mainly in an adhesion/cohesive failure mode after the joint is stripped (seen in Figure 11b).

### 4.2. Single-Lap FEM Stress Distribution

**Figure 12:**
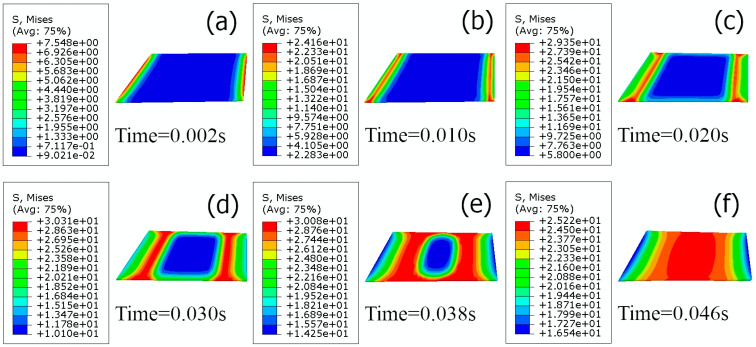
Stress distribution of the adhesive layer at different tensile–shear times. (**a**) Time = 0.002 s (**b**) Time = 0.01 s (**c**) Time = 0.02 s (**d**) Time = 0.03 s (**e**) Time = 0.038 s (**f**) Time = 0.046 s.

**Figure 13:**
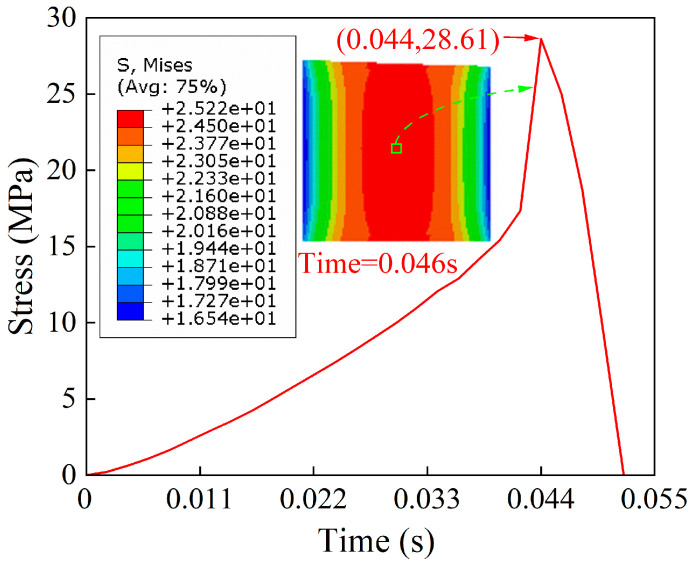
The stress from the center region element.

The stress distribution of the adhesive at different tensile–shear times is shown in Figure 12, and it can be found that the location of the maximum stress moves from the lap edge to the center region as time increases. The stress was extracted from the center region element at 0.046 s (seen in Figure 13), and it was discovered that the maximum stress was 28.61 MPa at 0.044 s.

**Figure 14:**
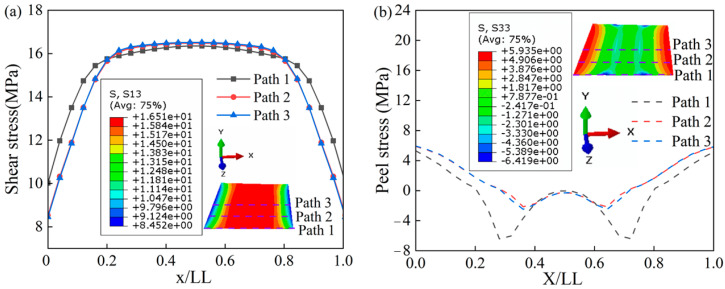
Stress distribution along three paths. (**a**) Shear stress; (**b**) peel stress.

At 0.044 s, path 1, path 2, and path 3 were created sequentially from the edge to the center region of the adhesive layer. The extracted path data are normalized (the length of the adhesive layer in the x-direction needs to be divided by LL), and the distribution of shear stresses on the three paths can be seen in Figure 14a. A symmetric distribution of shear stresses is shown as (x/LL = 0.5), with smaller shear stresses at the two ends and larger shear stresses at the center region. The maximum shear stress increases slightly from the edge to the center region (from path 1 to path 3). Similarly, the peel stresses for the three paths are shown in Figure 14b. The maximum peel stresses in the center region of the adhesive layer are close to the position “0”, part of the region has negative stress under compressive conditions. The larger stress change in the adhesive layer near the end of the lap region indicates the end of the joint’s occurring stress concentration. The same phenomenon has been shown in the literature [36], which is caused by the rotation of the adherend [37]. The maximum peel stress increases slightly from the edge to the center region. In addition, peel stresses and shear stresses provide further evidence of joint failure behavior.

### 4.3. Bonding Performance Prediction Based on Xgboost ML Algorithm

A total of 630 sets of single-lap FEMs with different process parameters were established, and the maximum load corresponding to each model was extracted for calculating the TSS values. Among the 630 sets of FEMs, LL increases from 10 to 30 mm, and the increment is 1 mm. TAL increases from 0.1 to 1 mm, and the increment is 0.1 mm. Roughness values are 1.16 μm, 0.89 μm, and 0.76 μm.

**Figure 15:**
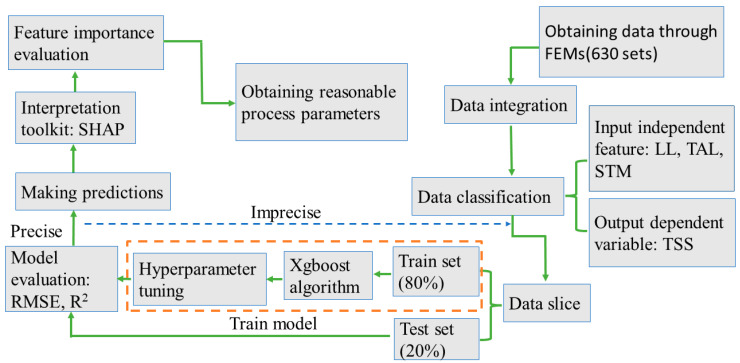
The flow chart for predicting the metal–metal bonding performance based on the Xgboost ML algorithm.

The flow chart for predicting the metal–metal bonding performance based on the Xgboost ML algorithm is shown in Figure 15. The process can be described as:

(1) A total of 630 sets of TSS values with different process parameters were obtained from 630 sets of single-lap FEMs and then merged.

(2) Data classification: the input independent features are LL, TAL, and roughness, and the output variables are TSS values.

(3) Data slicing. The training set is 80% of the total dataset, and the test set is 20%. The Xgboost model was trained from 504 sets of training set data, and 126 sets of test set data were input to the Xgboost model for TSS prediction.

(4) The best modeling parameters for the Xgboost model were obtained by performing hyperparameter optimization.

(5) The importance ranking of the input features on the model output was evaluated with SHAP, and the reasonable bonding process parameters were optimized.

Generally, a larger value of R^2^ (near 1) and a smaller RMSE (close to 0) mean that the model is more precise, and the calculated equations for RMSE and R^2^ are shown in Equations (10) and (11), respectively.

(10) $$RMSE=\sqrt{\frac{1}{N}\sum_{i=1}^{N}{\left(y_{ai}-y_{pi}\right)}^{2}}$$

(11) $$R^{2}=1-\frac{\sum_{i=1}^{N}{\left(y_{pi}-y_{ai}\right)}^{2}}{\sum_{i=1}^{N}{\left(y_{ai}-\bar{y_{a}}\right)}^{2}}$$

where *N* denotes the total number of samples, $y_{ai}$ indicates the true values, $y_{pi}$ represents the predicted values, and $\bar{y_{a}}$ means the average of all real values.

**Figure 16:**
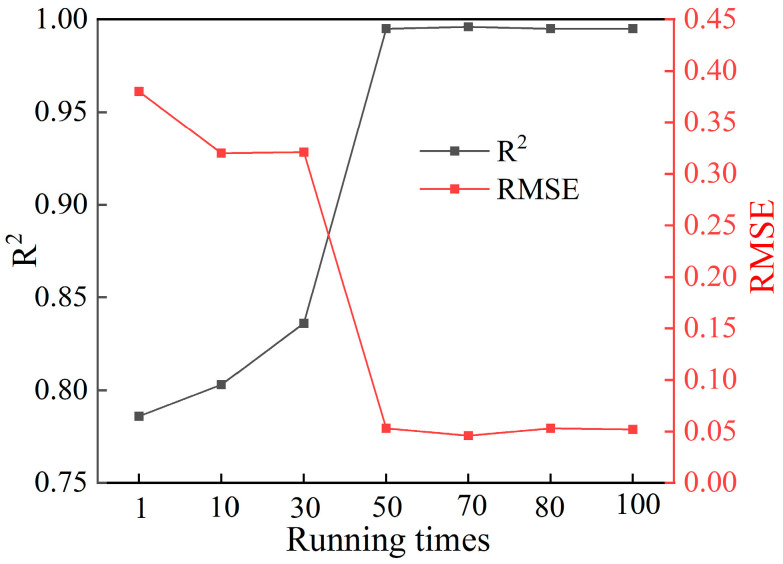
The change graphs of RMSE and R^2^ values with the running times of the Xgboost model.

**Figure 17:**
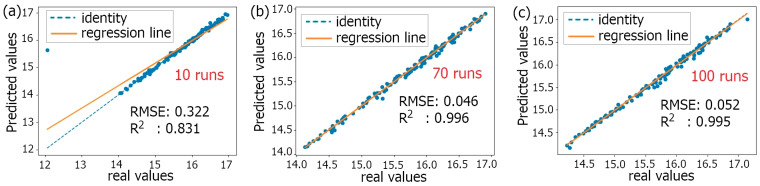
Comparison of predicted TSS and true TSS values: (**a**) 10 runs; (**b**) 70 runs; (**c**) 100 runs.

The program was written and debugged in the Jupyter Notebook interface of Anaconda3 software. The model accuracy indicators were assessed with the regression coefficient (R^2^) and the root mean square error (RMSE). The model’s prediction is accurate if the RMSE is closer to 0 and the R^2^ is closer to 1. Otherwise, the dataset was re-sliced to train the Xgboost model until the model was accurate. In order to obtain a more accurate prediction model, and combined with the computational efficiency of existing computers, the Xgboost model performed 100 runs in this work (shown in Figure 16). After 50 runs, the RMSE and R^2^ values of the model were more stable. Strikingly, based on the values of R^2^ and RMSE, the Xgboost model with 70 runs can achieve better prediction results. Figure 17 compares the predicted TSS and the true TSS under different running times with the Xgboost model, where the blue dashed line serves as the baseline, and the orange solid line represents the regression line fitted based on the predicted and the real TSS data points. The real TSS denotes the TSS calculated based on single-lap FEM. The model’s prediction results are accurate when the regression line coincides well with the baseline.

**Figure 18:**
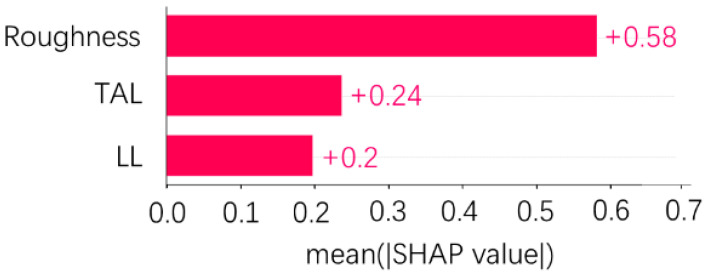
The importance ranking graph of input features on model output.

**Figure 19:**
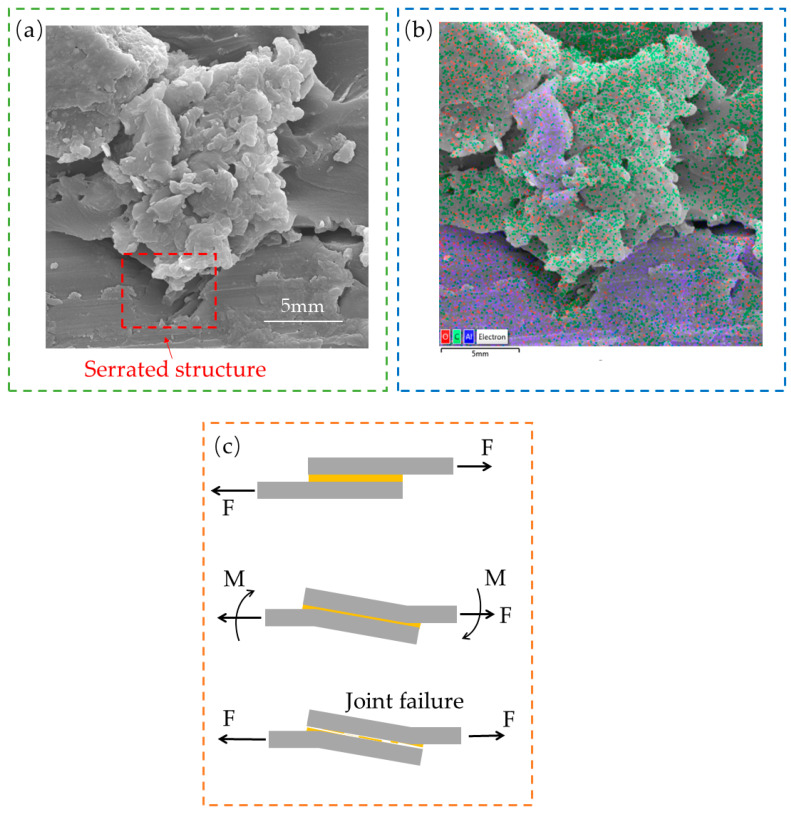
Schematic diagram of interfacial bonding and bending moment. (**a**) Interfacial bonding; (**b**) EDS analysis; (**c**) bending moment.

The Xgboost model with 70 runs was selected and combined with SHAP to determine the importance ranking of process parameters on TSS (shown in Figure 18). SHAP regards all features as contributors for each sample and produces a prediction value for each feature. The SHAP values are additive, and the SHAP values of the features can be calculated using Equation (12). The SHAP values for roughness, TAL, and LL were 0.58, 0.24, and 0.2, respectively. It means that the importance ranking from high to low is roughness, TAL, and LL, and the symbol “+” indicates that the feature positively influences the model output. Firstly, a porous structure was formed after the pretreatment of the metal surface. The porous structure is serrated when viewed in cross-section (shown in Figure 19a). The porous structure improves the wettability and mechanical occlusion between the adherend and the adhesive [38], and EDS analysis of the interface between the metal and the adhesive of the fractured specimen in Figure 11b (shown in Figure 19b) shows that Al, O, and C elements are mainly present at the interface, and the porous structure formed after anodization is Al_3_O_2_ [39]. Al-O-C chemical bonds were formed at the interface [14], thereby improving the interfacial bonding. Surface microscopic morphology is mainly characterized by the average roughness Ra, and the roughness affects the wetting angle [40]. Surface microscopic morphology decides the mechanical properties of the SLJs [41]. Thus, roughness has the highest importance ranking for TSS. Secondly, the change in TAL largely affects the degree of eccentricity of the load in the tensile–shear process. The transmission of the load is affected and then generates the bending moment (shown in Figure 19c). TSS decreases as TAL increases, so the TAL has a higher importance ranking for TSS. Finally, when the LL is long enough, the increase in LL has little effect on the strength of the joint [12], which means that the effect of LL on TSS has some limitations, so LL has the lowest importance ranking for TSS. In addition, the sensitivity analysis of LL and TAL on shear load is conducted using a single factor in the literature [42], and it was found that TAL has a higher sensitivity to shear loads than LL.

(12) $$y_{i}=y_{base}+f(x_{i}^{\left(1\right)}+\ldots +x_{i}^{\left(j\right)}+\ldots x_{i}^{\left(k\right)})$$

where $f(x_{i}^{\left(j\right)})$ denotes the SHAP value of the *j*-th feature for the *i*-th sample; $y_{base}$ represent the baseline value of SHAP and is the average of all sample prediction sets; and $y_{i}$ is the predicted value of the *i*-th sample.

**Figure 20:**
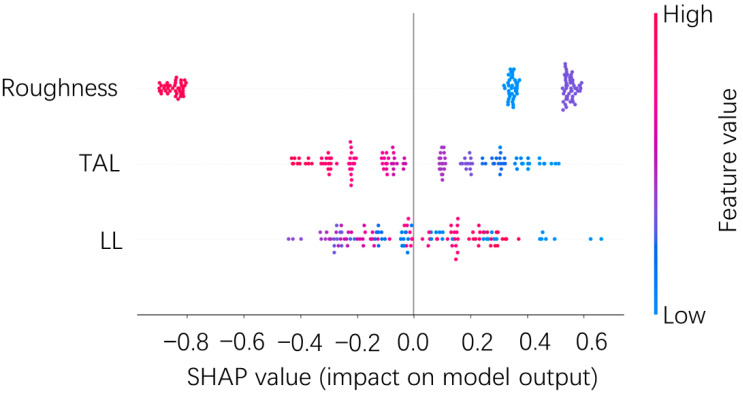
Global interpretation diagram.

In order to understand more intuitively whether each data sample in each feature has a positive or negative effect on the model output by the global interpretation diagram (seen in Figure 20), it can be observed that the SHAP value first increases and then decreases as the roughness value increases, indicating that medium roughness was favorable for improving TSS. Medium roughness will increase the wettability and mechanical occlusion of the adhesive with the adherend and improve the bonding performance. A very rough surface is challenging to be entirely impregnated with resin [43]. Smaller roughness makes it harder to form a robust mechanical occlusion between the adhesive and the adherend. The SHAP value increases as the TAL decreases, implying that a thinner TAL is more favorable for improving TSS, and the results are the same as in the literature [10]. The TAL increase affects the transfer of load, and the shear stress was gradually transformed into a mixture of shear and peel stress, increasing the bending moment and weakening the strength of the joint, whereas, with the increase in LL, it did not show that the SHAP value kept increasing all the time. Among them, some of the smaller LL SHAP values are the highest, indicating that the longer LL can increase the maximum load and thus increase the TSS. However, the much longer LL joints increase the risk of being peeled off and have a lower TSS. Meanwhile, the literature [44] stated that the peak load does not increase infinitely with LL and will reach a limit.

**Figure 21:**
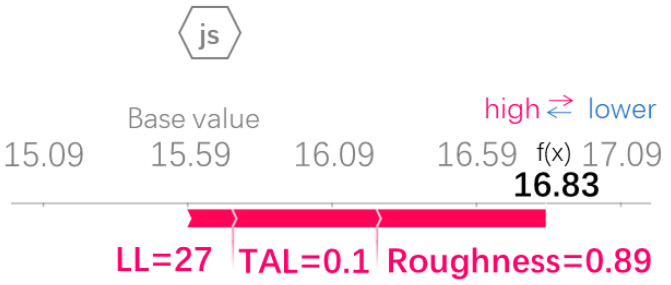
The influence graph of the features on the prediction results.

**Figure 22:**
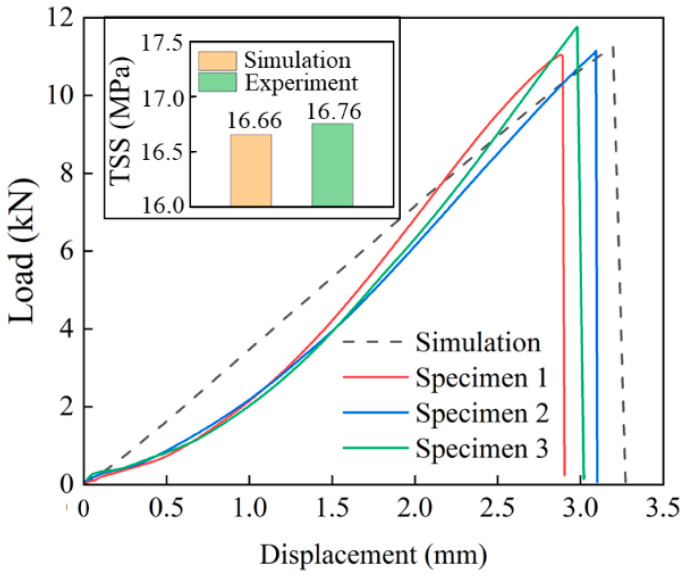
The verification of ML prediction results.

SHAP decomposed the final prediction into the sum of the contributions of all input variables with Equation (13) [45], and the effect of the input features on the prediction results can be quantified in Figure 21. The base value (15.59 MPa) represents the average values of the prediction for the whole training set, the features determine the deviation between the prediction and the base value, and the length of the bar indicates the degree of the contribution of the features to the prediction value. When LL is 27 mm, TAL is 0.1 mm, and roughness is 0.89 μm, the final predicted value is 16.83 MPa, which is close to the TSS values calculated from the single-lap FEM (the FEM from the ML-optimized parameters) and process experiment (shown in Figure 22), which are 16.66 MPa and 16.76 MPa, respectively. The validity of the Xgboost algorithm prediction results was proved. It is worth noting that the load–displacement curves of the FEM and process experiments in Figure 22 show some deviation in the loading stage because the single-lap specimen is clamped with a hydraulic fixture rather than being hand-tightened. After the upper end of the single-lap specimen is clamped, the hydraulic fixture will produce an upward force when clamping the lower end of the specimen, and the specimen is in a short compression state under the action of the upper and lower hydraulic fixtures, causing the curve in the loading stage fitting with a small amount of deviation. As the tensile–shear process goes on, the pressure on the specimen from the upper and lower hydraulic fixtures is released, and the curve fit between the experiment and simulation becomes better and better, especially in the vicinity of reaching the maximum load.

In addition, the corresponding FEM from the ML-optimized parameters shows a maximum load of 11.25 kN in Figure 22, which is 20.8% higher than the maximum load (9.31 kN) of the FEM in Figure 9a. Meanwhile, the TSS (16.76 MPa) with the experiment measured in Figure 22 is 14% higher than the TSS (14.7 MPa) located in Figure 9b. It is feasible to use the Xgboost ML algorithm to optimize process parameters and predict the bonding performance.

(13) $$g\left(x^{\prime }\right)=\varphi_{0}+\sum_{i=1}^{M}\varphi_{i}x_{i}^{\prime }$$

where $x^{\prime }$ denotes the vector of simplified input variables obtained from the original input variable *x* in the dataset; *M* indicates the number of features in the dataset; *φ_0_* means a constant when all inputs are zero; and *φ_i_* denotes the attribute value for each feature *i*.

## 5. Conclusions

In this work, for the purpose of improving the phenomenon of the adhesive layer seeping out from the lap edges, the SLJ specimen was prepared by PCPs and the SCC process. The bonding performance of metal–metal SLJs was evaluated using process experiments, single-lap FEM, and the Xgboost ML algorithm. Based on the interpretation toolkit SHAP, the process parameters (LL, TAL, and roughness) were arranged according to the importance ranking on the bonding performance, and reasonable process parameters were determined. The following are the key findings:

(1) The single-lap FEM was verified by a process experiment, and the simulated maximum load and TSS error were less than 1.5%.
(2) When the LL ranged from 10 to 30 mm, the TAL ranged from 0.1 to 1 mm, and the roughness measurements were 1.16 μm, 0.89 μm, and 0.76 μm, respectively. Medium roughness, thinner TAL, and longer LL were favorable for improving TSS, and the importance ranking of process parameters on TSS from high to low is roughness, TAL, and LL.
(3) The optimized values of LL, TAL, and roughness were 27 mm, 0.1 mm, and 0.89 μm, respectively. The experimentally measured TSS values increased by 14% from the optimized process parameters via the Xgboost ML model.

This work still has some limitations, as only process experiments, FEM, and machine learning methods were used to optimize the metal-to-metal bonding process parameters and improve the bonding performance. Future research should focus on the performance and failure mechanism of metal–metal joints under special conditions, such as hot and humid environments, mildew, and other environments, and further promote the broader engineering applications of metal–metal joints.

## Figures and Tables

**Table 1:** Metal surface pretreatment process.

Type	Surface Pretreatment	Mean Value of Roughness/μm
Type-1	400# sandpaper sanding followed by anodizing	1.16
Type-2	800# sandpaper sanding followed by anodizing	0.89
Type-3	1200# sandpaper sanding followed by anodizing	0.76

**Table 2:** Mechanical properties of the 2A12-T4 aluminum alloy.

Tensile Strength/MPa	Yield Stress/MPa	Young’s Modulus/GPa	Elongation/%
442	260	18.0	23–26

**Table 3:** DW-3 adhesive mechanical parameters.

Roughness/μm	*E* (GPa)	*G*1 = *G*2 (GPa)	$t_{n}^{0}$ (MPa)	$t_{s}^{0}=t_{t}^{0}$ (MPa)	$G_{n}^{C}$ (J/mm^2^)	$G_{S}=G_{T}$ (J/mm^2^)
1.16 0.89 0.76	5	7.6 8.339 9.149	8	17 19 18.6	9	16.5 18 17.8

where *E*, *G*1, and *G*2 represent the elastic modulus, tangential 1 modulus, and tangential 2 modulus of the adhesive layer unit, respectively. $t_{n}^{0}$, $t_{s}^{0}$, and $t_{t}^{0}$ mean the maximum critical stresses of the cohesive unit in the normal direction, tangential direction 1, and tangential direction 2, respectively. $G_{n}^{C}$, $G_{S}$, and $G_{T}$ are the normal, tangential, and mixed fracture energy, respectively.

## Data Availability

The data in this study are available from the corresponding author upon reasonable request.

## List of Acronyms and Abbreviations

**Abbreviation**	**Full name**
SLJs	Single-lap joints
FEMs	Finite element methods
ML	Machine learning
SHAPs	Shapley additive explanations
TSS	Tensile–shear strength
TAL	Thickness of the adhesive layer
LL	Lap length
GP	Gel point
PCP	Pre-curing process
SCC	Secondary complete curing
FBG	Fiber Bragg grating
R^2^	Regression coefficient
RMSE	Root mean square error
